# Phenotypic and genotypic drug resistance profile of *Salmonella* serovars isolated from poultry farm and processing units located in and around Mumbai city, India

**DOI:** 10.14202/vetworld.2018.1682-1688

**Published:** 2018-12-16

**Authors:** R. N. Waghamare, A. M. Paturkar, V. M. Vaidya, R. J. Zende, Z. N. Dubal, A. Dwivedi, R. V. Gaikwad

**Affiliations:** 1 Department of Veterinary Public Health, Bombay Veterinary College, Parel, Mumbai, Maharashtra, India; 2 Department of Veterinary Public Health, ICAR - Indian Veterinary Research Institute, Izatnagar, Bareilly, Uttar Pradesh, India

**Keywords:** multidrug-resistant, poultry, *Salmonella* spp, tetracycline

## Abstract

**Background and Aim:**

The extensive use of antimicrobials in poultry has led to an increase in bacterial multidrug resistance, and the emergence of multidrug-resistant nontyphoidal *Salmonella* is a global problem. This study was performed to detect antibiotic-resistant *Salmonella* serovars in poultry farming and processing environment.

**Materials and Methods:**

A total of 956 various samples, comprising 432 farm origin, 324 poultry processing stage wise and environmental, and 154 product processing stages and environmental samples, were collected from poultry farms and processing units located in and around Mumbai city. Of a total of 71 recovered isolates, 42 randomly selected *Salmonella* isolates were subjected for antibiotic susceptibility testing by disk diffusion method and serotyping. A total of 31 serotypically confirmed isolates were characterized for the presence of *tetA, tetB, bla*_TEM_, and CTX-M gene.

**Results:**

Higher resistance was recorded against Doxycycline (100%), followed by Oxytetracycline (97.62%), Neomycin (88.10%), Erythromycin (83.33%), Tetracycline (78.57%), and Ceftizoxime (35.71%). Resistance from 0.00 to 26.19 percent was found to antimicrobials, namely Norfloxacin (26.19%), Ampicillin (21.43%), Azithromycin (21.43%), Ciprofloxacin (19.05%), Colistin (4.76%), Streptomycin (16.67%), Cefotaxime (14.19%), Enrofloxacin (14.29%), Amoxyclav (14.29%), Gentamicin (7.14%), Chloramphenicol (4.76%), Amikacin (4.76%), and Ceftazidime (0.0%). Results demonstrate that the *Salmonella* Virchow dominated and all serotypes were found to carry Tetracycline resistance gene *tetA*, 5 isolates were found to be positive for *bla_TEM_*, whereas none of the isolates were carrying *tetB* and CTX-M gene.

**Conclusion:**

This study revealed that there is a significant rise of Tetracycline resistance with the presence of *tetA* gene in *Salmonella* spp. which indicates selective pressure for adopting resistance against tetracycline group of antibiotics.

## Introduction

Foodborne risk factors for human health can be recognized from poultry which includes microbiological and chemical risks, wherein *Salmonella* spp. contamination and residues from veterinary medications are important risks [1]. Microbiological risk factors are so prevailing that they can be found in almost all systems of poultry production [2]. Poultry and poultry products are known reservoirs for these foodborne pathogens, and numerous reports described the prevalence of *Salmonella* associated with live poultry, production environments, and processing plants [3]. *Salmonella* has been a pathogen of significance and is a major cause of gastroenteritis in humans [4]. *Salmonella* illness has linked with exposure to meat; a review of the Centers for Disease Control and Prevention 2012 outbreak data indicated that 10 out of 25 outbreaks were related to live poultry, shell eggs, or further processed poultry products [5].

Salmonellosis in animal and human may occur due to the involvement of >2500 serovars [6]. In India, *Salmonella* Virchow, *Salmonella* Typhimurium, and *S*. Enteritidis are reported as major nontyphoidal *Salmonella* serovar from poultry [7]. Among veterinary residues, antibiotic residues in meat have been a rising issue in recent years in India. Antibiotics have been used in poultry for the treatment of infections and also to counteract the adverse consequences of stress responses [8]. The presence of antimicrobial residues in meat has several impacts on health aspects to the consumer like possible contribution to the development of antibiotic resistance bacteria [9]. Various workers reported drug resistance genes against tetracycline and broad- and extended-spectrum β-lactamase antibiotics in *Salmonell*a due to selective pressure [10].

Therefore, the purpose of the present study was to examine the presence of multidrug-resistant (MDR) *Salmonella* spp. in poultry farming and processing establishment. The increasing single and multiple antimicrobial-resistant *Salmonella* strains isolated from human cases of Salmonellosis have been associated with widespread use of antimicrobial agents in food animal production [11]. This may clearly represent a public health risk by transfer of resistant *Salmonella* strains to humans through the consumption of contaminated poultry products.

## Materials and Methods

### Ethical approval

Since no animals were used in this study, ethical approval was not needed.

### Sampling and study period

In the present study, for isolation of *Salmonella* spp., a total of 956 various samples, comprising 432 poultry farm origin, 324 poultry processing stage wise and environmental, and 154 product processing stages and environmental samples, were collected from poultry farms and processing units located in and around Mumbai city during December 2015-December 2017. Bacterial isolates were isolated by IS 5887 (Part 3): 1999 [12]. Bacterial isolates were identified on the basis of cultural characteristics on BGSA and XLD media, Gram staining, and conventional biochemical test. The isolates were further characterized by *invA* gene as per the method of Rahn *et al*. [13], to identify pathogenic *Salmonella* spp. Isolates identified as *Salmonellae* were sent to Poultry Diagnostics and Research Center, Loni Kalbhor, Pune, for serotyping.

### Antimicrobial susceptibility testing

Randomly selected 42 *Salmonella* spp. isolates were tested for antimicrobial susceptibility by disk diffusion method (Kirby–Bauer test) using the following randomly selected 19 antimicrobials which were commonly used in animal and humans (HiMedia Laboratories, Pvt., Ltd., Mumbai, India): Gentamicin (10 μg), Azithromycin (15 μg), Ceftizoxime (30 μg), Amikacin (30 μg), Amoxyclav (30 μg), Norfloxacin (10 μg), Oxytetracycline (30 μg), Enrofloxacin (10 μg), Ciprofloxacin (5 μg), Streptomycin (10 μg), Colistin (10 μg), Chloramphenicol (30 μg), Cefotaxime (30 μg), Ceftazidime (30 μg), Ampicillin (10 μg), Neomycin (30 μg), Erythromycin (15 μg), Tetracycline (30 μg), and Doxycycline (30 μg). According to the Clinical and Laboratory Standards Institute [14] guidelines and interpretative criteria and based on inhibition zone, the isolates were categorized as resistance (R), intermediate (I), and sensitive (S).

### Detection of antimicrobial resistance genes

The presence of genes associated with resistance to Tetracycline (*tetA* *and tetB*), broad-spectrum β-lactamases (*bla*_TEM_), and β-lactamases with extended spectrum (CTX-M) in confirmed 31 serotypes were detected by polymerase chain reaction (PCR) as per the methods [15-17] depicted in Table-1.

**Table-1:** Standardization of PCR method for genotypic characterization of *Salmonella* spp.

S. No.	Gene name	Target	Primer Sequence (5’-3’)	Thermal profiles for PCR	Product Size (bp)	Reference
1	*invA*	Invasion-associated protein	F: GTGAAATTATCGCCACGTTCGGGCAA	94°C×2 m/94°C×30 s - 65°C×60 s - 72°C×120 s (30 cycles)/72°C×5 m	284	[13]
			R: TCATCGCACCGTCAAAGGAACC			
2	*bla*_TEM_	Broad-spectrum β-lactamases	F: ATGAGTATTCAACATTTCCG	95°C×5 m/95°C×60 s - 55°C×60 s - 72°C×60 s (35 cycles)/72°C×7 m	867	[15]
			R: CTGACAGTTACCAATGCTTA			
3	*tetA*	Tetracycline	F: GCTACATCCTGCTTGCCTTC	95°C×5 m/95°C×60 s - 64°C×30s - 72°C×30 s (40 cycles)/72°C×10 m	210	[16]
			R: CATAGATCGCCGTGAAGAGG			
4	*tetB*	Tetracycline	F: TTGGTTAGGGGCAAGTTTTG	95°C×5 m/95°C×60 s - 64°C×30s - 72°C×30 s (40 cycles)/72°C×10 m	659	[16]
			R: GTAATGGGCCAATAACACCG			
5	CTX-M	β-lactamases with extended spectrum	F: ATGTGCAGYACCAGTAARGTKATGGC	95°C×5 m/94°C×30 s - 62°C×90 s - 72°C×60 s (40 cycles)/72°C×10 m	593	[17]
			R:TGGGTRAARTARGTSACCAGAAYCA GCGG			

PCR: Polymerase chain reaction

## Results

### Occurrence of *Salmonella* spp.

On screening these 956 samples, 71 positive *Salmonella* spp. were recovered with the occurrence of 7.4%. All the isolates were further confirmed as *Salmonella* spp. by amplification of *invA* gene.

### Susceptibility testing through agar disk diffusion method

**Figure-1:**
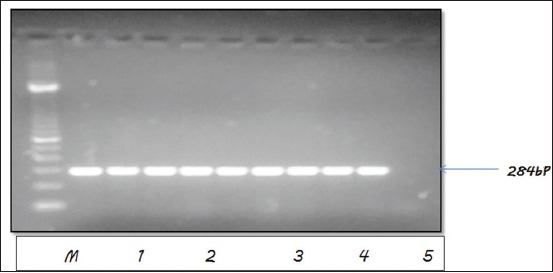
Polymerase chain reaction assay for the detection of virulence gene *invA* of *Salmonella* isolates. Lane M: 100 bp standard DNA ladder. Lane 1-9: Isolates positive for invA gene. Lane 10: Negative control.

Antibiotic susceptibility testing was performed for randomly selected 42 confirmed *invA* gene-positive isolates (Figure-1) with 19 frequently used antibiotics stated earlier. All of the isolates from this study were found to be resistant to more than two antibiotics.

### Antimicrobial resistance pattern

The PCR assay (*invA* gene) positive 42 *Salmonella* isolates were tested against 19 commonly used antimicrobials for resistance pattern. Higher resistance was recorded against Doxycycline (100%), followed by Oxytetracycline (97.62%), Neomycin (88.10%), Erythromycin (83.33%), Tetracycline (78.57%), and Ceftizoxime (35.71%). Resistance was also found to routinely used antimicrobials, namely Norfloxacin (26.19%), Ampicillin (21.43%), Azithromycin (21.43), Ciprofloxacin (19.05%), Streptomycin (16.67%), Cefotaxime (14.19%), Enrofloxacin (14.29%), and Amoxyclav (14.29%). All the isolates were found susceptible to Ceftazidime. The drugs found to be effective in terms of susceptibility were Colistin (83.33%), Chloramphenicol (95.24%), Gentamicin (88.10%), and Amikacin (95.24%). Results are depicted in Table-2.

**Table-2:** Phenotypic antimicrobial resistance pattern of *Salmonella* spp.

S. No.	Antimicrobial agent		Susceptibility of *Salmonella* isolate (%)		

			Sensitive	Intermediate	Resistance
1	Gen10	Gentamicin	88.10	4.76	7.14
2	AZM15	Azithromycin	52.38	26.19	21.43
3	CZX30	Ceftizoxime	23.81	40.48	35.71
4	AK30	Amikacin	95.24	0.00	4.76
5	AMC30	Amoxyclav	83.33	2.38	14.29
6	NX10	Norfloxacin	64.29	9.52	26.19
7	O30	Oxytetracycline	2.38	0.00	97.62
8	Ex10	Enrofloxacin	59.52	26.19	14.29
9	CIP5	Ciprofloxacin	64.29	16.67	19.05
10	S10	Streptomycin	66.67	16.67	16.67
11	CL10	Colistin	83.33	0.00	16.67
12	C30	Chloramphenicol	95.24	0.00	4.76
13	CTX30	Cefotaxime	54.76	30.95	14.29
14	CAZ30	Ceftazidime	100.00	0.00	0.00
15	AMP10	Ampicillin	78.57	0.00	21.43
16	N30	Neomycin	11.90	0.00	88.10
17	E15	Erythromycin	0.00	16.67	83.33
18	TE30	Tetracycline	21.43	0.00	78.57
19	DO30	Doxycycline	0.00	0.00	100.00

### Serotyping of isolates

Out of 42 isolates, 31 were identified as *S*. Virchow (20), *Salmonella* Newport (6), and *S*. Typhimurium (5), whereas 11 isolates remained untypable, this indicates the dominance of serotype *S*. Virchow in poultry farming and processing system under study.

### Charactering of serotypes for the presence of *tetA, tetB, bla_TEM_*, and CTX-M genes

**Figure-2:**
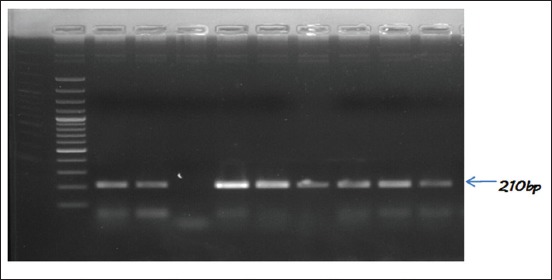
Polymerase chain reaction assay for detection of *tetA* gene of *Salmonella* isolates. Lane 1: TrackIt™ 100bp DNA ladder. Lane 2: Positive control (NIFL1). Lane 3: Positive sample. Lane 4: Negative control. Lanes 5-10: Positive samples.

**Figure-3:**
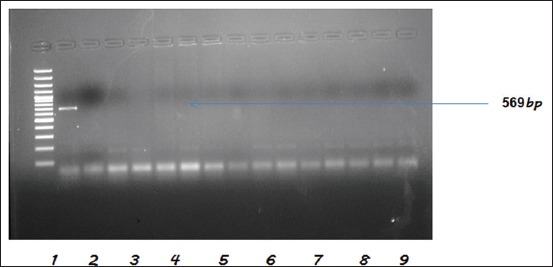
Polymerase chain reaction assay for the detection of *tetB* gene of *Salmonella* isolates. Lane 1: TrackIt™ 100bp DNA ladder.Lane 2: Standard positive control of *Salmonella* spp. Lane 3: Negative control. Lanes 4-16: Negative samples.

**Figure-4:**
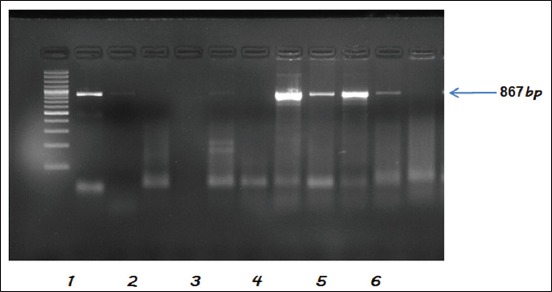
Polymerase chain reaction assay for the detection of *bla*_TEM_ gene of *Salmonella* isolates. Lane 1: TrackIt™ 100bp DNA ladder. Lane 2: Positive control (NIFL1). Lanes 3-7: Negative samples. Lanes 8-11: Positive samples. Lane 12: Negative samples.

All the tested *Salmonella* serotypes (n=31) were found to carry Tetracycline resistance gene *tetA*, whereas none of them were carrying *tetB* gene. Whereas 5 isolates were found positive for *bl*a_TEM_ indicating resistance against Broad spectrum β-lactamases, and none of the isolate was found to be carrying CTX-M gene (Table-3 and Figures 2-4). The *bla*_TEM_ gene was observed in two isolates from each of *S*. Typhimurium (NIWH6 and ACWH6) and *S*. Newport (SCDM4 and RCKS4), while one isolate of *S*. Virchow (RCPD4). The overall occurrence of Tetracycline resistance and broad-spectrum β-lactamases resistance in *Salmonella* isolates was 100 and 16.12%, respectively.

**Table-3:** Antibiotic resistance and virulence marker gene detected in different *Salmonella* serotypes.

S. No.	Source of isolate	Sample code	Serogroup	Antibiotic resistance marker				Virulence marker *invA*

				*bla*_TEM_	*tetA*	*tetB*	CTX-M	
1	Cloacal swab	NICS9	*S*. Virchow	-	+	-	-	+
2	Litter	NIFL1	*S*. Newport	-	+	-	-	+
3	Litter	NIFL9	*S*. Virchow	-	+	-	-	+
4	Feces	NIF8	*S*. Virchow	-	+	-	-	+
5	Drinker	NIDR7	*S*. Virchow	-	+	-	-	+
6	Drinker	NIDR6	*S*. Typhimurium	-	+	-	-	+
7	Worker hand	NIWH6	*S*. Typhimurium	+	+	-	-	+
8	Litter	PIL1	*S*. Virchow	-	+	-	-	+
9	Feces	PIF7	*S*. Virchow	-	+	-	-	+
10	Drinking water	PIDW5	*S*. Typhimurium	-	+	-	-	+
11	Worker hand (evisceration)	RCWH3	*S*. Virchow	-	+	-	-	+
12	Carcass contact platform	RCCP5	*Salmonella* Newport	-	+	-	-	+
13	Chopping board	RCCB1	*S*. Virchow	-	+	-	-	+
14	Chopping board	RCCB3	S. Virchow	-	+	-	-	+
15	Knife	RCKS4	*S*. Newport	+	+	-	-	+
16	Post defeathering	RCPD4	*S*. Virchow	+	+	-	-	+
17	Post defeathering	RCPD3	*S*. Virchow	-	+	-	-	+
18	Post evisceration	RCPE2	*S*. Virchow	-	+	-	-	+
19	Post evisceration	RCPE3	*S*. Virchow	-	+	-	-	+
20	Post evisceration	RCPWC2	*S*. Virchow	-	+	-	-	+
21	Neck skin of eviscerated bird carcass	RCNE2	*S*. Virchow	-	+	-	-	+
22	Defeathering machine	SCDM4	*S*. Newport	+	+	-	-	+
23	Worker hand (evisceration)	SCWH4	*S*. Virchow	-	+	-	-	+
24	Chopping board	SCCB5	*S*. Typhimurium	-	+	-	-	+
25	Knife swap	SCKS4	*S*. Virchow	-	+	-	-	+
26	Post defeathering	SCPD2	*S*. Virchow	-	+	-	-	+
27	Post evisceration	SCPE4	*S*. Virchow	-	+	-	-	+
28	Neck skin of eviscerated bird carcass	SCNC4	*S*. Virchow	-	+	-	-	+
29	Worker hand (evisceration)	ACWH6*	*S*. Typhimurium	+	+	-	-	+
30	Deboning cone	ACDC6	*S*. Newport	-	+	-	-	+
31	Raw meat	FPRM1	*S*. Newport	-	+	-	-	+
Total				05(16.12)	31(100)	00	00	31

* Isolate (ACWH6) received gene bank accession number NCBI GenBank MG844415. S. Virchow= *Salmonella* Virchow, S. Typhimurium=*Salmonella* Typhimurium, S. Newport=*Salmonella* Newport

## Discussion

Poultry and their environments are act as the major sources of foodborne salmonellosis to human beings. In the present study, an overall occurrence of *Salmonella* isolation from poultry samples and environment was 7.4% which is of public health significance.

Although antimicrobials have a distinct advantage in the management of infection and promotion of growth in broilers, indiscriminate and non-judicious extensive use of antimicrobials could lead to the emergence of antimicrobial resistance. This uncontrolled use of drugs may exert selective pressure and promotes the proliferation of drug-resistant strains of *Salmonella* in poultry production system [18]. This was coupled with poor environmental sanitation and workers personal hygiene in processing could be a potential threat to public health. This study demonstrated that *Salmonella* isolates could able to acquire resistance. Resistance pattern varied from isolates to isolates, but 100.00, 97.62, 88.10, and 83.33% isolates showed resistance to Doxycycline, Oxytetracycline, Neomycin, and Erythromycin, respectively. These resistant isolates in the poultry environment might work as a potential reservoir for transfer of resistant genes into other highly infectious Gram-negative pathogens present in the poultry environment.

Similar results were reported by Ishihara *et al*. [19] with respect to Oxytetracycline (82.0%). However, neomycin resistance pattern has comparable finding with Carramiñana *et al*. [20] who reported 53.4% resistance to neomycin, while in contrast to the findings of Poppe *et al*. [21] who observed resistance <2% in *Salmonella* isolates.

In the present study, resistance <30% was recorded to antimicrobials, namely Norfloxacin, Ampicillin, Azithromycin, Ciprofloxacin, Colistin, Streptomycin, Cefotaxime, Enrofloxacin, Amoxyclav, Gentamicin, Chloramphenicol, Amikacin, and Ceftazidime. In the study, *Salmonella* isolates were found resistant to Ampicillin (21.43%), Amoxyclav (14.29%), and Cefotaxime (14.19%) which is a rising concern for India because increased use of β**-**lactam antibiotics to treat enteric infection, *Salmonella* spp. might be acquiring resistance to third-generation cephalosporin antibiotic in different parts of the world and leading to clinical treatment failure [22]. Isolates were susceptible to Amikacin (95.24%), Ceftazidime (100.00%), and Chloramphenicol (95.24%) suggested that limited use and effective control by farmers on these compounds are associated with high susceptibility.

Scur *et al*. [23] found approximately 55% of resistance to Amoxicillin plus Clavulanic acid, Cefotaxime, Ceftazidime, Amikacin, and Norfloxacin, while Ghazaey and Mirmomeni [24] observed 70–100% resistance to Gentamicin, Cefotaxime, Streptomycin, and Amoxicillin. Abunna *et al*. [25] observed 100% sensitivity to gentamicin and ciprofloxacin, whereas Ghoddusi *et al*. [26] observed it to Ampicillin, Cefazolin, Cefotaxime, Cefixime, Ceftriaxone, Enrofloxacin, Difloxacin, and Gentamicin which is found not in accordance with the observations of the present study.

Various serovars such as *Salmonella* Enteritidis, *S*. Typhimurium, *S*. Virchow, and *S*. Newport are important nontyphoidal causes of human salmonellosis caused by consumption of contaminated poultry products [26]. More than 53 serovars have been reported from India, and this number is on ever increasing. Various research workers isolated similar serotypes from poultry farm and processing environment, but the occurrence of *S*. Virchow (65.66) in the current study is higher than the previously isolated report [27]. Similar findings were reported by Khanna [7] who found *S*. Virchow (48%) and *S*. Typhimurium (24%), followed by *S*. Infantis (13%), *S*. Indiana (7%), *S*. Enteritidis, and *S*. Hadar (4% each) in samples collected from retail meat shops in New Delhi. Recently, research workers isolated *S*. Newport from chicken meat [28,29]. Report of *S. Newport* in a poultry farm and processing environment is a serious issue, and rapid rise of MDR *Salmonella* serovar Newport isolates over the past decade as important causes of human Salmonellosis [30].

Several serotypes are consistently found at a higher incidence, and the distribution of *Salmonella* serotypes from poultry sources varies geographically and changes over a period of time [31]. The high rates of serogroup *S*. Virchow in our studies taken together with previous data may suggest that this serogroup may be more adapted to poultry farm and processing environments under study. A total of 11 isolates remained untypable which were positive by PCR assay for *invA* gene but negative by serotyping has been termed as untypable. This may be attributed to the presence of rough mutant strains which lack the specific side chains responsible for “O” specificity or some additional abnormalities of the core structure [28].

All the tested *Salmonella* serotypes were found to carry Tetracycline resistance gene *tetA* (100%) whereas none of them were carrying *tetB* gene. Whereas, 5 isolates were found positive for *bla*_TEM_ (16.12%) and none of the isolate was found to carry CTX-M gene. The prevalence of broad-spectrum β-lactamases resistance (*bla*_TEM_) in the serovars from poultry farm and processing units was not uniformly distributed in samples analyzed. For *bla*_TEM_ gene, two isolates were positive from each of *S*. Typhimurium (NIWH6 and ACWH6) and *S*. Newport (SCDM4 and RCKS4), while one isolate was positive for *S*. Virchow (RCPD4). Uniform distribution of phenotypic Tetracycline resistance (Doxycycline and Oxytetracycline) among all the serovars along with the presence of *tetA* gene indicates selective pressure on the *Salmonella* spp. for adopting resistance against Tetracycline group of antibiotics.

The overall occurrence of Tetracycline resistance and broad-spectrum β-lactamases resistance in *Salmonella* isolates was 100 and 16.12%, respectively. The findings in the present study regarding *tetA* and *tetB* gene were in corroboration with Soufi *et al*. [10]. On the contrary Lebdah *et al*. [32] who reported 70 and 20%, prevalence for *tetA and tetB*, respectively .

The *tetA* gene associated with tetracycline efflux pumps was reported to be predominant in *Salmonella*, and *Escherichia coli* isolates from livestock and food animals, and it may present in mobile elements and is acquired by bacteria through horizontal gene transfer [33,34]. Result confirms good phenotypic and genotypic correlation for Tetracycline resistance among *Salmonella* isolates.

The findings for *bla*_TEM_ gene in the current study are supported by the results of other researchers [35]. The cefotaximases (CTX-M-type extended-spectrum beta-lactamases) have become the most widespread β-lactamases over the past few years. In the current study, none of the serotypes was found to be positive for gene encoding CTX-M. Our report is in agreement with Wittum *et al*. [36], whereas our results are contradicting with reports of Riano *et al*. [37], who reported *Salmonella* serovars with the CTX-M gene, which might be due to the influence of the genomic environment on local dissemination of resistance genes among different bacterial genera [38] and antimicrobials that could not be extensively used among poultry which may confer selective pressure to acquire resistance.

Kodimalar *et al*. [39] reported that occurrence of tetracycline residue in feed samples reflects the extensive use of chlortetracycline agent in chicken production systems. Van Boeckel *et al*. [40] reported, in Asia, that antimicrobial consumption in chicken is expected to grow by 129% by 2030, wherein currently in India the issue of overuse of these antibiotics is of particular significance with South Coast of India, while the cities of Mumbai and Delhi are becoming antimicrobial consumption hotspots.

In the present study, phenotypic resistance to Tetracycline group of antibiotics with the presence of *tetA* gene is in correlation. This indicates selective pressure on *Salmonella* isolates leading to an increase in the prevalence of Tetracycline resistance posing a risk to human and animal health. However, drug-susceptible *Salmonellae* can also become resistant by acquiring drug resistance plasmids from other enteric pathogens in the intestinal tract of patients [41].

Our results confirm dissemination of multidrug-resistant *S*. Virchow, *S*. Typhimurium, and *S*. Newport from farm to processing environment which may pose a serious risk to human health. These serotypes may affect human by causing nontyphoidal Salmonellosis. Information on daily dose, duration of treatment, number of animals treated, and consumption data may be useful to relate the simultaneous existence of antimicrobial resistance [42]. In the present study, unfortunately, supportive information regarding antimicrobial usage of various other antimicrobials was not available; therefore, it was not possible to associate the observed resistances to use of antimicrobials.

## Conclusion

This study revealed a significant rise in Tetracycline resistance with presence of *tet*A gene in *Salmonell*a spp. indicating selective pressure for adopting resistance against tetracycline group of antibiotics. Dissemination of multidrug-resistant *S*. Virchow, *S*. Typhimurium, and *S*. Newport from farm to processing environment may pose a serious risk to human health, and these serotypes may affect human by causing nontyphoidal Salmonellosis.

## Authors’ Contributions

RNW designed the experiment under the supervision of AMP. RJZ and RVG supervised work. Media preparation, sample collection, and bacteriological analysis were performed by RNW and VMV. Molecular work was performed by RNW, ZND, and AD. All authors participated in the draft and revision of the manuscript. All authors read and approved the final manuscript.
